# The COVID-19 pandemic and the menstrual cycle: research gaps and opportunities

**DOI:** 10.1093/ije/dyab239

**Published:** 2021-12-02

**Authors:** Gemma C Sharp, Abigail Fraser, Gemma Sawyer, Gabriella Kountourides, Kayleigh E Easey, Gemma Ford, Zuzanna Olszewska, Laura D Howe, Deborah A Lawlor, Alexandra Alvergne, Jacqueline A Maybin

**Affiliations:** 1 Population Health Sciences, Bristol Medical School, University of Bristol, Bristol, UK; 2 MRC Integrative Epidemiology Unit, University of Bristol, Bristol, UK; 3 NIHR Bristol Biomedical Research Centre, Bristol, UK; 4 School of Anthropology, University of Oxford, Oxford, UK; 5 Translational Health Sciences, Bristol Medical School, University of Bristol, Bristol, UK; 6 Institut des Sciences de l'Évolution, Université de Montpellier, Montpellier, France; 7 MRC Centre for Reproductive Health, University of Edinburgh, Edinburgh, UK

## Menstrual cycle features and the COVID-19 pandemic

Over 50% of the global population will experience menstruation, and menstrual disorders are extremely common and debilitating.^1^ Problematic menstruation may cause anaemia,^2^ has a significant negative impact on quality of life and is a huge socioeconomic burden for women, their families, health services and society.^3–7^ Standardized parameters for typical menstruation have been defined by the International Federation of Gynecology and Obstetrics (FIGO) regarding menstrual frequency, duration, regularity and volume, and deviation from these may constitute abnormal uterine bleeding.^8^ Features of the menstrual cycle are also increasingly being recognized as ‘vital signs’—acting as both indicators and possible determinants of broader health and well-being.^9^ For example: irregular and long menstrual cycles have been associated with a greater risk of premature mortality,^10^ and infrequent or absent menstruation can be an indicator of reduced fertility,^3^ which itself can be associated with a number of chronic conditions.^11^ Since the beginning of the COVID-19 pandemic, there have been accumulating discussions on social media and blogs indicating that women have experienced menstrual changes, including altered menstrual duration, frequency, regularity, and volume (heavier bleeding and clotting), increased dysmenorrhoea and worsened premenstrual syndrome (PMS) (e.g. Morgan 2021^12^). More recent anecdotal reports of menstrual changes after vaccination for COVID-19 have fuelled vaccine hesitancy or refusal. There is an important public health imperative for accurate scientific investigation of these phenomena.

Unfortunately, questions about menstruation have been excluded from most large-scale COVID-19 studies (including vaccine trials), so it is currently unclear how many women have experienced menstrual cycle changes, how long these changes persisted, whether menstrual changes reflect common and expected fluctuation in menstrual features over time or the impact of an exposure (e.g. pandemic restrictions, infection/illness, treatment, vaccine) and what exactly this exposure is. Given this complexity, the impact of any menstrual changes since the start of the pandemic is also unclear. Even outside the context of COVID-19, studying menstrual cycle features is challenging. Normal variation exists within women over the lifespan and between women in relation to characteristics such as history of infertility, parity, body mass index (BMI) and exercise.^13^ In addition, menstrual cycle features such as volume, pain and PMS symptoms are subjective and data are necessarily collected, in health care as well as research, by self-report.

**Figure 1:**
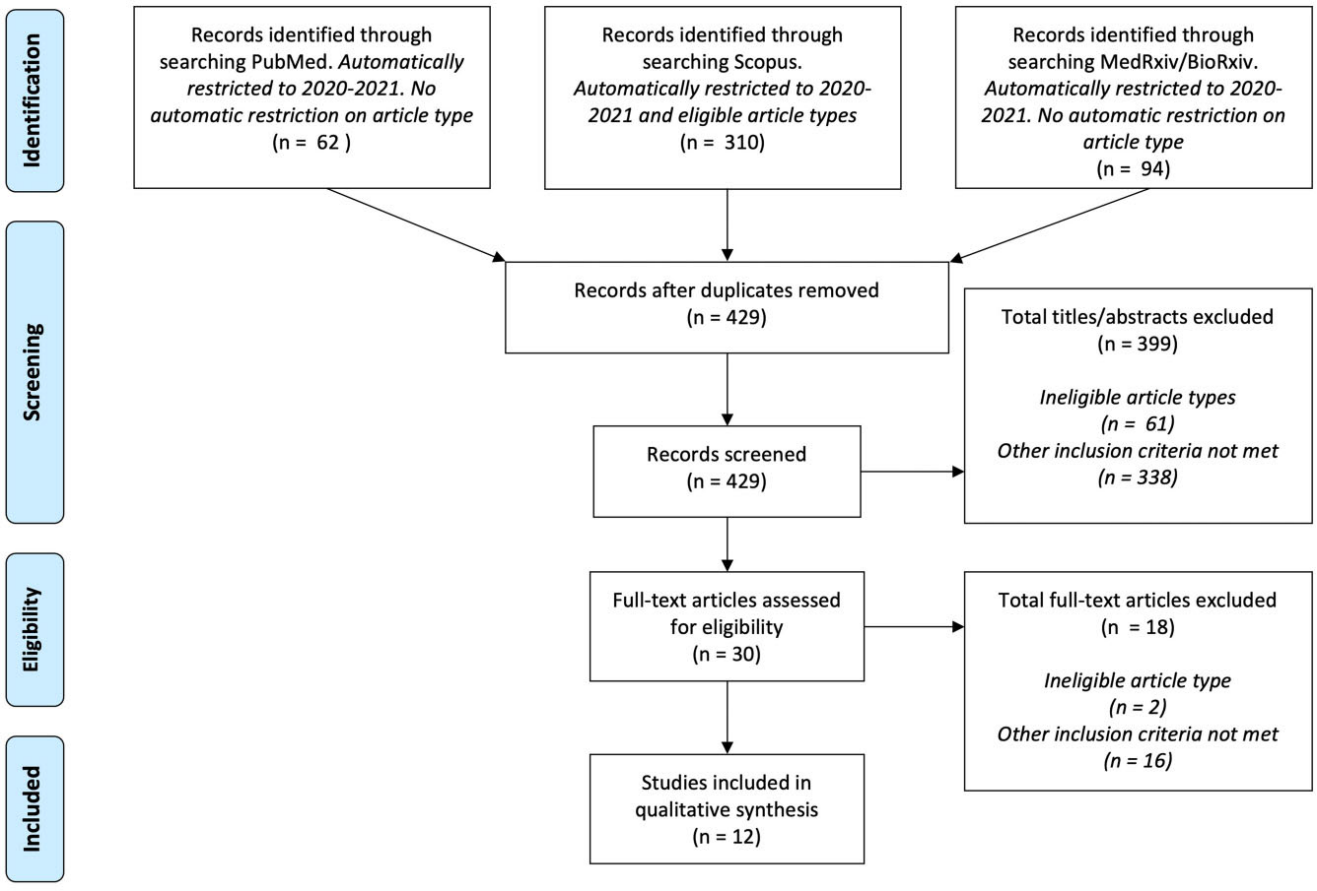
A flowchart of articles identified and filtered through our systematic review protocol

In this paper, we aimed to identify and evaluate the existing scientific literature on menstrual cycle feature changes in the COVID-19 pandemic and provide suggestions for future research. Using a pre-specified search protocol (available at [https://osf.io/xg3mw/], developed in accordance with PRISMA guidelines^14^) we searched PubMed, Scopus and preprint servers (BioRxiv and MedRxiv) across all fields for terms related to menstruation AND COVID-19 (full review methods in the Supplementary Methods, available as Supplementary data at *IJE* online). The review process is summarized in Figure 1. We identified 12 small studies reporting on menstrual cycle features during the pandemic, either in relation to the COVID-19 pandemic period (Table 1; ^15–23^) or COVID-19 illness specifically (Table 2; ^24–26^) All studies are summarized in more detail in Supplementary Table S1, available as Supplementary data at *IJE* online. Articles were included if they described features of the menstrual cycle (e.g. cycle length, regularity, heaviness, pain, PMS symptoms) before and during, or over the course of, the COVID-19 pandemic. The final search was performed on 31 August 2021.

**Table 1:** A summary of studies comparing menstrual cycle features during and before the COVID-19 pandemic (see Supplementary Table S1, available as Supplementary data at *IJE* online for more information)

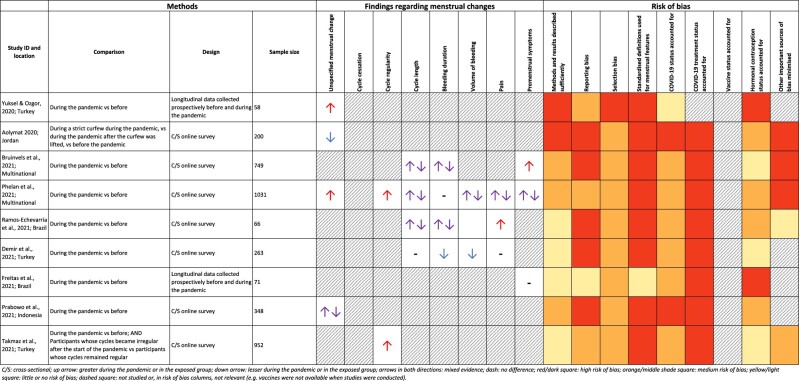

**Table 2:** A summary of studies comparing menstrual cycle features in COVID-19 cases or controls, or before vs during illness (see Supplementary Table S1, available as Supplementary data at *IJE* online for more information)

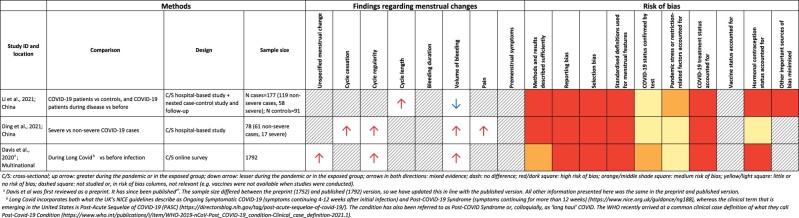

## Possible causal explanations

**Figure 2:**
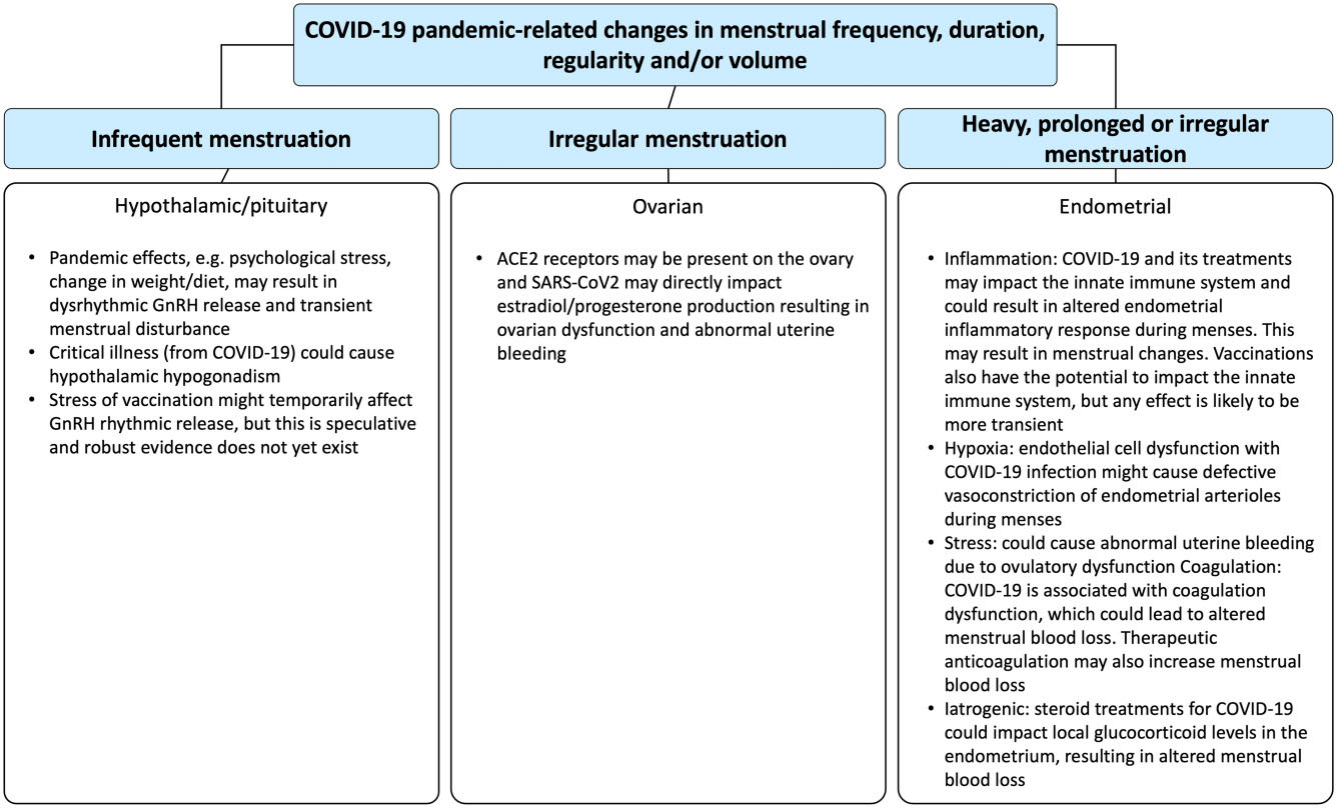
How the pandemic mitigation/control factors and COVID-19 illness, treatment and vaccines may impact on the hypothalamic-pituitary-ovarian-endometrial axis to alter menstrual frequency, duration, regularity and/or volume^29–32^

The menstrual cycle is regulated by a complex interplay of hormones that interact with the immune, vascular and coagulation systems, and these interactions can influence menstrual bleeding and severity of (pre)menstrual symptoms.^28^ These changes will occur following effects on hypothalamic-pituitary-ovarian-endometrial function (Figure 2). In the context of the COVID-19 pandemic, it is plausible that such effects could be instigated by one or more of the following exposures.

### COVID-19 mitigation and control strategies

COVID-19 mitigation and control strategies, like lockdowns and social distancing, have led to increases in psychological stress, depression and anxiety and reductions in general well-being, particularly in young adults and women.^33^ Studies have also identified associations between the pandemic and weight gain and changes in health behaviours.^34^ Psychological stress is a known risk factor for hypothalamic hypogonadism, resulting in infrequent or absent menstruation.^35^ There is also a well-known link between changes in weight and the menstrual cycle, and some (inconclusive) evidence that the menstrual cycle can be altered by health behaviours such as changes in alcohol consumption, diet and physical activity.^36^

The studies in Table 1 consider menstrual cycle feature changes in relation to the pandemic period. Their results could therefore reflect effects of pandemic mitigation and control strategies (assuming they are not explained by confounding or other types of bias).

### SARS-CoV-2 infection and COVID-19 illness

SARS-CoV-2 infection and COVID-19 illness could affect the hypothalamic-pituitary-ovarian-endometrial axis with resulting changes to the menstrual cycle. Hypothalamic hypogonadism may occur in the presence of any severe illness, including COVID-19, and result in temporary amenorrhoea or infrequent menses. This protective mechanism enables diversion of energy resources from reproduction to the immune response.^37^ This may also explain why those experiencing long-term symptoms of Ebola infection (Post Ebola Syndrome; possibly analogous to Long Covid) have reported menstrual cessation or irregularity.^38^

Alternatively, or additionally, there may be more specific interactions between the reproductive system and SARS-CoV-2 infection. This may occur at the ovarian/endometrial level. The ovarian hormone progesterone is predominantly anti-inflammatory.^39^ Progesterone levels fall dramatically before menstruation, with a resulting influx of inflammatory cells to the local endometrial environment that ultimately leads to the shedding of the functional endometrium at menstruation.^40^ Intense vasoconstriction of the specialized endometrial spiral arterioles and activation of the local coagulation system act to limit menstrual blood loss. It is proposed that ACE2 receptors are present on ovarian and endometrial tissue^41^^,^^42^ and hence SARS-CoV-2 infection may hypothetically affect ovarian hormone production and/or the endometrial response at menses. For example, altered endometrial leukocyte number/phenotype during or following SARS-CoV-2 infection has the potential to impact on menstrual blood loss. Previous research has shown that viral infection-induced immune disruption was associated with exacerbation of progesterone-related premenstrual symptoms.^43^ Furthermore, COVID-19 has also been associated with endothelial cell dysfunction and alterations in the coagulation system, both critical components of endometrial function at menstruation, indicating a potential endometrial mechanism for menstrual disturbance.^40^^,^^44^

Reciprocally, reproductive hormones and/or menstruation may also affect the severity of COVID-19, i.e. variation in severity of symptoms of COVID-19 across different stages of the menstrual cycle.^45^ In a recent study of Long COVID symptoms by Davis *et al.*^27^ (identified through our review and summarized in Table 2), over a third of participants experienced relapses of symptoms during or before menstruation, i.e. during the most inflammatory phases of the cycle. Cyclical variation in symptoms has also been documented in those with myalgic encephalomyelitis/chronic fatigue syndrome (ME/CFS), a chronic condition often triggered by infection, which has been compared with Long Covid. Female ME/CFS patients often notice flare-ups of their symptoms during the premenstrual phase of their cycles or at the onset of menopause.^46^

The three studies in Table 2 consider menstrual cycle feature changes in relation to COVID-19 illness.

### COVID-19 treatments

COVID-19 treatments that have been advised for COVID-19 symptoms include antipyretics and analgesics, such as paracetamol, aspirin and other non-steroidal anti-inflammatories (NSAIDs). NSAIDs affect prostaglandin synthesis and endometrial prostaglandin levels, and via these mechanisms can reduce menstrual pain and blood loss.^47^^,^^48^ Although some observational studies suggest aspirin may alter blood loss, randomized controlled trials suggest this is not the case, though it does reduce pain associated with menstruation.^48^^,^^49^ For hospitalized COVID-19 patients, one of the earliest identified effective treatments was dexamethasone which may affect menstrual cycle patterns and blood loss through cortisol actions.^50^ The impact of novel (as opposed to repurposed) treatments, including the new monoclonal antibodies, is unknown, though anti-tumour necrosis factor (TNF) monoclonal antibodies have some impact on the endometrium in non-human primate studies.^51^ In addition, some treatments might cancel out cycle changes; for example, low oxygen saturation has been linked to low-grade inflammation and anovulatory cycles,^52^ but the possibility that mechanical ventilation might impact on cycles has not been explored.

We did not identify any studies of menstrual cycle feature changes in relation to COVID-19 treatments.

### COVID-19 vaccines

COVID-19 vaccines have been reported in the media and via government monitoring systems as causing changes in menstrual features (for example, as of early September 2021, more than 30 000 reports had been made to the UK Medicines and Healthcare Products Regulatory Agency’s yellow card surveillance scheme^53^). However, we did not identify any studies that explored this. Previous research on HPV and flu vaccinations have shown these can be associated with changes in menstrual cycle features.^54^^,^^55^ In a study of 30 000 Japanese women, receiving an HPV vaccine was associated with increased age-adjusted odds of hospital visits for ‘abnormal amount of menstrual bleeding’ [odds ratio (OR): 1.43, 95% confidence interval (CI): 1.13–1.82], ‘irregular menstruation’ (OR: 1.29, 95% CI: 1.12–1.49) and chronic, persisting ‘abnormal amount of menstrual bleeding’ (OR: 1.41, 95% CI: 1.11–1.79).^54^ In addition, a clinical study found that women had lower levels of post-ovulation progesterone following inactivated influenza vaccination.^55^ These studies suggest that there is an impact of vaccination (in general) on menstrual symptoms. Given the widespread use of vaccinations, we expect any effects to be transient. The mechanism by which vaccine effects on menstrual cycle features occur remains under -researched and undetermined.

## Potential research bias

In addition to true causal effects, it is also possible that observed associations between the COVID-19 pandemic and menstrual cycles could be explained by various types of bias. These include reporting bias introduced by heightened health awareness, stemming from people monitoring their own health more closely during the pandemic/illness and consequently being more likely to notice menstrual cycle features and report (apparent) changes compared with pre-pandemic/illness. This type of bias is possible even in hospital-based studies for some menstrual cycle features, like pain and PMS, which are measured subjectively. Many of the studies we identified relied on self-reported, often retrospective or cross-sectional data.

Studies have also relied on small, selected samples, meaning their findings may not be generalizable to other populations (i.e. lacking external validity) and are highly susceptible to selection bias, which could bias estimates (i.e. lacking internal validity). For example, online surveys select for internet users and are more likely to be completed by and shared among people affected by the condition being studied. This may result in biased estimates as well as limit the generalizability of findings to larger populations.

Furthermore, because of confounding, some studies are unable to distinguish between the various pandemic-related exposures (i.e. mitigation/control strategies, infection/illness, treatments, vaccines). In studies of the effect of COVID-19 illness on menstrual cycle features, another source of confounding could be hormonal contraceptive use, which is highly prevalent among women of reproductive age. Depending on the type of contraceptive, hormonal contraceptive use alters hormonal cyclicity, which can influence both menstrual cycle features and immune cyclicity, and therefore could potentially affect COVID-19 symptom severity. Some authors have claimed that estradiol, a component of the combined hormonal contraceptive pill, offers a protective effect against COVID-19 severity and mortality,^56^ and a study using data from the COVID Symptom Tracker App reported that women aged 18–45 taking the combined oral contraceptive pill had lower rates of predicted COVID-19 and lower hospital attendance than women taking no form of hormone therapy^57^ (although the role of residual confounding, most notably by age, cannot be ruled out). Hormonal contraceptive use might also be an effect modifier, for example pandemic-related exposures might affect features of contraceptive-controlled menstrual cycles differently from those of natural cycles. Thus, the relationship between COVID-19 severity and features of the menstrual cycle between hormonal contraceptive users and non-users, as well as between users taking progesterone only and combined hormonal contraceptives, merits further investigation. Four of the 12 studies in Tables 1 and 2 either stratified by hormonal contraceptive use or excluded users, four studies just described use within the sample and four did not mention it at all.

Finally, only one of the studies included here used a recognized questionnaire to measure menstrual cycle features (Freitas *et al.*^20^ used the Premenstrual Symptoms Screening Tool, PSST^58^) and no studies made full use of the globally agreed International Federation of Gynecology and Obstetrics (FIGO) standardized parameters for menstrual frequency, duration, regularity and volume.^8^ Using standardized parameters is important for the generation of robust, comparable research data, and for assessing whether menstrual changes deviate from what would be considered normal and expected variation in physiological menstruation. As has been highlighted in the COVID-19 research more widely, there is also a need to use common definitions of COVID-19 and Long Covid.^59^ Adherence to such recommendations during study design will increase consistency and facilitate the interpretation of results to drive clinical and societal impact.

## The need for further research

Although new research is continuously being published on COVID-19, the lack of high-quality research focusing on COVID-19 and the menstrual cycle mirrors the broader focus of medical research, which does not prioritize women’s health, particularly outside the context of pregnancy. The finding that menstrual cycles appear to have been affected by the COVID-19 pandemic could have important implications for society, gender-based inequalities and the post-COVID economic recovery. Women are more likely than men to have significant childcare responsibilities and insecure employment and finances,^60^ and therefore are disproportionately affected by the COVID-19 pandemic^60^; 26% of the global population are women of reproductive age and the vast majority menstruate. This population is also of working age and/or in education. In addition to affecting women’s physical health, mental well-being and quality of life,^9^ menstruation-related symptoms are an important source of economic burden, through decreased productivity and increased absence from the workplace ^61^ and school.^62^ Exacerbation of menstrual symptoms during the pandemic may be further compounded by pandemic-related issues with living arrangements and privacy, access to and affordability of menstrual products and reduced availability and accessibility of sexual and reproductive health care services.^63^ Therefore, as the world continues to cope with and begins to recover from the COVID-19 pandemic, there is a need for further research to help understand and mitigate the impacts of the pandemic on menstrual health, which could help to minimize gender-based health and social inequalities. The pandemic has also highlighted the need for more research to inform a broader understanding of how external, environmental factors can influence the menstrual cycle, and how the menstrual cycle can interact with other aspects of health in a bidirectional manner.

## Outstanding research questions and considerations

In Table 3 we provide a list of outstanding questions that could be applied to studies of any menstrual cycle feature.

**Table 3:** Outstanding questions about the relationship between the COVID-19 pandemic and menstrual cycle features

Area of study	Specific questions
The prevalence and characteristics of women experiencing menstrual cycle feature changes during the COVID-19 pandemic	What proportion of women have experienced changes to their menstrual cycle features during the pandemic? Which features were affected? To what extent? Does the association vary by demographic factors like age, parity, ethnicity and socioeconomic position? Is it influenced by any other factors, like hormonal contraceptive use?
The effect of pandemic mitigation and control strategies on menstrual cycle features	Which pandemic-related stressors and behaviour changes are associated with changes to menstrual cycle features? What are the underlying biological mechanisms? How persistent are these effects; how long do menstrual cycle changes take to revert to normal? Can behaviour-related or stress-relieving interventions help to regulate menstrual changes?
The effect of COVID-19 illness on menstrual cycle features	Is the severity of COVID-19 and the types of other symptoms experienced associated with variation in changes in menstrual cycle features? What biological mechanisms explain this effect?
The effect of COVID-19 treatments on menstrual cycle features	How do COVID-19 treatments affect menstrual cycle features? Does timing or type of treatment make a difference? Does menstrual cycle stage affect treatment efficacy or side effects?
The effect of COVID-19 vaccines on features of the menstrual cycle	How do COVID-19 vaccines affect menstrual cycle features? Does timing or type of vaccine make a difference? Does menstrual cycle stage affect vaccine efficacy or side effects?
The long term or latent effect of any pandemic-related exposure (mitigation/control strategies, COVID-19 illness, treatment and vaccines) on reproductive health	Do these exposures affect timing of menarche (if prepubertal children are exposed) or the menopause? Are there effects on fertility (both achieving and maintaining a pregnancy)? How? What are the best strategies to mitigate any negative effects?
Factors that could modify effects of these pandemic exposures on menstrual health	How have affected people dealt with changes to their menstrual cycle features? Which self-care approaches have they used and how effective have these been? Have people experienced difficulties accessing support from health care providers? Have they had difficulty accessing menstrual products? (There have already been some studies on this.^63^) Which groups have been most affected? What are the most effective management strategies for health care providers to follow during a pandemic?
Reverse effects: the effect of menstrual cycle stage on COVID-19 and Long Covid symptoms	Are women more susceptible to coronavirus infection, or at higher risk of experiencing severe symptoms, at certain stages of the menstrual cycle (e.g. during the perimenstrual phase, when ovarian hormone levels are transiently but significantly decreased)? What are the underlying biological mechanisms? Do type and severity of acute COVID-19 or Long Covid symptoms show variation over the menstrual cycle? Does the menstrual cycle and/or female reproductive hormones explain the higher incidence of Long Covid in working-age women than in men? Does hormonal therapy or contraception affect symptoms? Does cycle stage influence the accuracy of COVID-19 tests? If Long Covid symptoms indeed fluctuate according to the menstrual cycle, what can this suggest about the pathophysiology of Long Covid itself?

Studies that minimize selection and reporting bias, control for key confounders (selected depending on the exposure and outcome of interest), and use harmonized or standardized definitions of menstrual cycle features and COVID-19/Long Covid, are needed. Researchers will need to consider whether they can feasibly disentangle and identify effects of the various exposures (pandemic mitigation/control factors, acute COVID-19, Long Covid, treatments, vaccines) for a growing proportion of the population who have been exposed to most or all of them. To be most informative for the global population of women and people who menstruate, future research should continue to focus on populations from a variety of countries and settings. Nine of the 12 studies in Tables 1 and 2 were conducted on populations from low- or middle-income countries (Brazil, China, Indonesia, Jordan and Turkey) and three were open to people from any country (although we note that high-income countries were over-represented). For their target populations, future studies should consider and adequately describe the situation with the pandemic (for example, which restrictions were in place, for how long, how they were enforced, general compliance etc.), social attitudes to menstruation, awareness of menstrual health and availability and accessibility of menstrual products and health services. These factors provide much needed social context to enable findings to be interpreted and compared across populations.

An ideal study would have prospective, repeat longitudinal data on menstrual cycle patterns and symptoms, in addition to repeat data on exposures related to pandemic mitigation and control, COVID-19 (SARs-Cov-2 infection, acute symptoms and symptoms of Long-Covid), treatments and vaccination. Setting up such a study this long after the start of the pandemic would not be ideal. However, it is possible that existing research cohorts or digital fitness trackers and menstrual cycle tracking smartphone apps may have already collected some of these repeat measures. Each of these methods has its own strengths and limitations. For example, data from smartphone menstrual cycle tracker apps are collected frequently and longitudinally on large numbers of women, but the data are affected by a high degree of missingness, and collected from a select group of smartphone users, many of whom are using the app to track fertility while trying to get pregnant. Collecting relevant data on menstrual cycle changes within existing cohort studies also enables longitudinal collection of data and missingness may be lower (and potential selection bias can be investigated with existing data), but frequency of repeat data collection and sample sizes are likely to be smaller than datasets collected from smartphone apps. Cautious interpretations from individual studies will be necessary. More conclusive inferences about the direction of any causal effect may be possible by triangulating evidence garnered using several different approaches with different and unrelated key sources of bias.^64^ An international research network could help address several of the research questions in Table 3. For example, any studies with repeat measures that could separate different exposures could be pooled to increase statistical power. Exploring heterogeneity between studies would help understanding of how context might moderate any effect of the various exposures, and could also help with understanding the extent to which results might be biased. We would urge anyone with such data to contact the authors to discuss the possibility of establishing such a network.

## Conclusion

Anecdotal evidence discussed online, government monitoring systems, and a small number of scientific studies of variable quality suggest that many women have experienced changes to features of their menstrual cycle during the COVID-19 pandemic, either due to pandemic-related factors like stress and behaviour changes and/or due to COVID-19 illness itself. Further research into the effects of COVID-19 and other health-related exposures on women’s menstrual health is urgently needed. Without robust menstrual data collection and analysis, menstrual problems will continue to be occult and under-managed in society. Determining the scale of menstrual problems, their cause and the impact on those who menstruate and wider society will allow identification of new preventative and therapeutic strategies. Findings can inform policies to mitigate against gender inequalities in health and society, allowing us to ‘build back better’ post-COVID.

## Supplementary Data

Supplementary data are available at *IJE* online.

## Funding

G.C.S., K.E., L.D.H., A.F. and D.A.L. work in the UK Medical Research Council Integrative Epidemiology Unit at the University of Bristol (MC_UU_00011/5 and MC_UU_00011/6), and A.F. and D.A.L. also work in the Bristol National Institute for Health Research (NIHR) Biomedical Research Centre. D.A.L. is a British Heart Foundation Chair (CH/F/20/90003) and NIHR Senior Investigator (NF-0616–10102). G.C.S. is financially supported by the Medical Research Council (New Investigator Research Grant, MR/S009310/1) and the European Joint Programming Initiative ‘A Healthy Diet for a Healthy Life’ (JPI HDHL, NutriPROGRAM project, UK MRC MR/S036520/1). J.M. is funded by a Wellcome Trust Fellowship (209589/Z/17/Z), RSE grant (1007), Tenovus Scotland and centre grants (G1002033 and MR/N022556/1). The funders had no role in designing this research.

## Author Contributions

G.C.S.: conceptualiation, manuscript writing. G.S., G.K., K.E., Z.O., G.F., J.M., A.A., L.D.H., A.F., D.A.L.: manuscript review and edit. J.M. and D.A.L.: clinical expertise.

## Conflict of Interest

None declared.

## Supplementary Material

dyab239_Supplementary_Data
