# Supplementary material for: The COVID-19 pandemic and the menstrual cycle: research gaps and opportunities
Source: Int J Epidemiol. 2021 Dec 2;51(3):691–700. doi: 10.1093/ije/dyab239 (PMC8690231; doi:10.1093/ije/dyab239)
Supplement: dyab239_Supplementary_Data [file dyab239_supplementary_data.zip › ije-2021-06-0898-File007.docx]

Systematic review methods

# Search strategy

We searched peer-reviewed academic literature repositories PubMed and Scopus, and the pre-print servers MedRxiv and BioRxiv. In all repositories, we searched across all fields using keywords related to the menstrual cycle (menstruation, menstrual) AND the COVID-19 pandemic (COVID-19, SARS-CoV-2, coronavirus, lockdown).

- In PubMed, we will search across all fields for: *menstrua* AND (COVID* OR "sars-cov*" OR "coronavirus" OR “lockdown”)*
- In Scopus, we will search across all fields for: *menstrua* AND (COVID* OR "sars-cov*" OR "coronavirus" OR “lockdown”)*
- In MedRxiv and BioRxiv (which can be searched simultaneously from the same advanced search function on either website) it is not currently possible to search using wildcards and Boolean operators, so we conducted multiple searches of each possible pairwise combination of keywords related to the menstrual cycle and COVID-19, i.e.:
  - COVID menstruation, SARS-COV-2 menstruation, coronavirus menstruation, lockdown menstruation, COVID-19 menstrual, SARS-COV-2 menstrual, coronavirus menstrual, lockdown menstrual

# Inclusion criteria

## Participants

Our population of interest is people who menstruate, including people on hormonal contraception who experience withdrawal bleeds. Studies that include only pre-menarchal or post-menopausal people were not eligible. There are no other geographical or demographic restrictions on the types of participants relevant to our review. Where participants were defined as having (had) COVID-19, we included studies with either confirmed (via RT-PCR/antigen test or antibody test) and/or suspected cases.

## Concept

The core concept examined by this review is changes to menstrual cycle features. Menstrual cycle features include cycle length, volume of blood, clotting, pain, irregular menstruation, amenorrhoea (cessation of menstruation) and the emotional and physical symptoms of premenstrual syndrome, including mood changes, irritability, breast pain, acne, and bloating. We included studies that described data on at least one menstrual cycle feature.

## Context

The context we are interested in is the coronavirus disease 2019 (COVID-19) pandemic. We are interested in the effects of COVID-19 itself (and treatment and vaccine effects) and pandemic-related stress and lifestyle changes. Therefore, eligible studies must describe or assess menstrual cycle features during the pandemic period, which we defined as starting in January 2020, in line with when the World Health Organisation first declared COVID-19 a Public Health Emergency of International Concern. At the time of writing (September 2021), the pandemic is ongoing, so there was no restriction on the end of the study period.

## Types of evidence sources and eligible study design

Our scoping review considered primary research articles, published in peer-reviewed academic journals or in academic pre-print servers. Reviews, commentaries, editorials, opinions, guidelines and protocols will be excluded. Conference abstracts were eligible for inclusion but none were identified. We placed no restriction on study design type (e.g., qualitative studies, case reports, trials, surveys, cohort studies and case-control studies are all eligible). However, all studies must have assessed menstrual cycle features in the same individuals over time (e.g. at different timepoints before/during/after lockdown restrictions, or before/during/after COVID-19 illness) or between groups of individuals differentially exposed to lockdown restrictions or pandemic-related stress or lifestyle changes, and/or differentially exposed to COVID-19 (i.e. COVID-19 cases vs controls).

# Source of evidence selection

Identified articles were downloaded to the reference manager Zotero and duplicates were removed by manually comparing information (authors, journal, title). Articles were screened against the eligibility criteria based on titles/abstracts by one author (Sharp). For papers that pass this first round of screening, one author (Sharp) accessed the full text versions and further evaluated the papers against the screening criteria. All decisions were checked with a second author (Sawyer). If there was any disagreement, a third author (Easey) was consulted.

# Data extraction

The following data was extracted: Author(s), year of publication, peer-review/pre-print status, aims/purpose, study design, comparison groups, population characteristics, sample size, menstrual features studied, pandemic/COVID related features studied, data collection dates, potential confounding factors and sources of bias, key strengths and limitations, key findings that relate to the review questions. All information was compiled in a spreadsheet. Data was extracted by one author (Sharp) and checked by another (Sawyer).

# Critical appraisal of individual sources of evidence

We had planned (and it is stated in our protocol) to use the Newcastle-Ottawa scales^1^ for case-control and cohort studies to assess the quality of each individual source. For trials, we had planned to use guidance from the Cochrane Handbook for Systematic Reviews, including the Cochrane risk-of-bias tool for randomized trials^2^.

However, we did not find any trials, and most studies were online surveys that did not fit the Newcastle-Ottawa scales well. Therefore, we assessed risk of bias in a more tailored way. We assessed bias under the following headings:

**Reporting bias:** this included relying solely on subjectively reported data and recall bias potentially introduced by retrospective report

**Generalisability and selection bias:** assessment of sample size, sample selection factors and sample characteristics in relation to the target population

**Confounding:** the ability of a study to identify the exposure causing menstrual changes, independent of any potentially confounding or other biasing factors. We considered the most important confounders (or modifiers/mediators) to be the various potential causal exposures: pandemic-related stress or restrictions, COVID-19 infection/illness, COVID-19 treatments; COVID-19 vaccines, as well as hormonal contraception use.

**Measurement of menstrual features and COVID-19:** whether studies used standardised/validated methods to define menstrual features, and whether COVID-19 cases were confirmed by PCR test (in studies where COVID-19 was the main exposure of interest)

**Scientific reporting:** the detail and coherence of the study methods and results as described in the paper

We scored risk of bias for each heading using a three-point system: high, medium, and little/no risk.

# References

1. Wells, G. *et al.* The Newcastle-Ottawa Scale (NOS) for assessing the quality of nonrandomised studies in meta-analyses.

2. Sterne, J. A. C. *et al.* RoB 2: a revised tool for assessing risk of bias in randomised trials. *BMJ* l4898 (2019) doi:10.1136/bmj.l4898.
